# Severe metabolic alkalosis and recurrent acute on chronic kidney injury in a patient with Crohn's disease

**DOI:** 10.1186/1471-2369-11-6

**Published:** 2010-04-18

**Authors:** Johannes Jacobi, Susanne Schnellhardt, Mirian Opgenoorth, Kerstin U Amann, Axel Küttner, Axel Schmid, Kai-Uwe Eckardt, Karl F Hilgers

**Affiliations:** 1 Department of Nephrology and Hypertension, University Erlangen-Nuremberg, Erlangen, Germany; 2 Institute of Pathology, University Erlangen-Nuremberg, Erlangen, Germany; 3 Institute of Radiology, University Erlangen-Nuremberg, Erlangen, Germany

## Abstract

**Background:**

Diarrhea is common in patients with Crohn's disease and may be accompanied by acid base disorders, most commonly metabolic acidosis due to intestinal loss of bicarbonate.

**Case Presentation:**

Here, we present a case of severe metabolic alkalosis in a young patient suffering from M. Crohn. The patient had undergone multiple resections of the intestine and suffered from chronic kidney disease. He was now referred to our clinic for recurrent acute kidney injury, the nature of which was pre-renal due to profound volume depletion. Renal failure was associated with marked hypochloremic metabolic alkalosis which only responded to high volume repletion and high dose blockade of gastric hypersecretion. Intestinal failure with stomal fluid losses of up to 5.7 litres per day required port implantation to commence parenteral nutrition. Fluid and electrolyte replacement rapidly improved renal function and acid base homeostasis.

**Conclusions:**

This case highlights the important role of gastrointestinal function to maintain acid base status in patients with Crohn's disease.

## Background

Short bowel syndrome is a rare and devastating complication in chronic inflammatory bowel disease following functional or anatomic loss of extensive segments of the small intestine [1]. The incidence is estimated at 2-3 per million [2]. The most common causes of short-bowel syndrome in adults include Crohn's disease, mesenteric ischemia, trauma, radiation enteritis and recurrent intestinal obstruction. The clinical characteristics of short bowel syndrome are defined by malabsorption, diarrhea, steatorrhea, fluid and electrolyte disturbances, and malnutrition [3]. Patients are at risk to develop short bowel syndrome if the length of viable small intestine is less than 200 cm. Under such conditions the absorptive capacity and intestinal adaptation is profoundly compromised and often necessitates total parenteral nutrition. With the advent of new immunosuppressive regimens intestinal transplantation has become the main therapeutic option in patients with irreversible intestinal failure. Current 1-year graft survival rates are as high as 80-90% [4].

## Case Presentation

A 27-year-old malnourished male (BMI 14.8 kg/m^2^) was referred to our hospital for recurrent acute kidney injury. The patient had been discharged a month earlier with an episode of acute kidney injury. Chronic kidney disease stage III was known since 2005, a renal biopsy performed in our clinic during his last hospitalization revealed FSGS-like glomerular lesions as well as acute tubular necrosis with mild oxalate deposits. Past medical history was remarkable for the diagnosis of Crohn's disease at age 17. As part of complicated inflammatory bowel disease the patient underwent subtotal colectomy in 2001, multiple resections of the small intestine between 2005 and 2008, as well as rectum extirpation in 2008. The latter procedure required placement of a terminal ileostoma. Profound diarrhea and hypersecretion as well as two seizures due to electrolyte disturbances following surgical procedures in 2005 required temporary home parenteral nutrition via a port catheter. Following adaptation the port system was removed. The patient had been on systemic as well as local corticosteroids and mesalazine for his medical condition in the past. Since fall 2008 the patient was solely treated with biweekly injections of adalimumab.

Vital signs upon presentation were as follows: blood pressure 80/40 mmHg, heart rate 100 beats/min., respiratory rate 13/min., temperature 36.8° Celsius. Physical examination was remarkable for cachexia, malnutrition and severe dehydration. In addition skin pallor and nasolabial dermatitis was noted. Initial laboratory tests were remarkable for normochrome and normocytic anemia (haemoglobin 8.6 g/dl) and acute kidney injury (creatinine 7.16 mg/dl, urea 117 mg/dl). Liver function and clotting tests were normal, total protein was 63.5 g/l, albumine 35.9 g/l. An arterial blood gas revealed severe hypochloremic metabolic alkalosis with partial respiratory compensation, the elevated anion gap further suggested metabolic acidosis (pH 7.56, pO_2 _80 mmHg, pCO2 58.2 mmHg, bicarbonate 52 mmol/l, sodium 133 mmol/l, potassium 2.6 mmol/l, chloride 65 mmol/l, anion gap 16.4). The patient believably denied episodes of vomiting or use of diuretics.

Urinalysis was only remarkable for alkaluria (pH 8.5) and moderate proteinuria (1.1 g/g creatinine), the urinary sediment was devoid of calcium oxalate crystals.

Due to the electrolyte imbalance further analysis of electrolyte concentration within the urine and stomal fluid was performed (table 1). Urinary chloride excretion was negligible at 6 mmol/l whereas potassium excretion was high at 113 mmol/l. With a serum and urine osmolality of 281 and 310 mosm/kg respectively, the transtubular potassium gradient was ~39 suggesting renal potassium wasting due to secondary hyperaldosteronism. Renin concentration was markedly elevated at 144 pg/ml (normal range: 3-28), plasma aldosterone was only slightly elevated at 176 pg/ml (normal range: 30-160) most likely due to the fact that blood samples were taken two days after admission when intensive potassium supplementation had already been initiated. Low urinary chloride excretion suggested extrarenal chloride loss. Indeed, stomal fluid losses were as high as 5700 ml per day and fecal chloride excretion was substantial at 87 mmol/l (table 1).

**Table 1:** Blood, urine and stomal fluid pH and electrolyte concentration

Parameter	Blood	Urine	Stomal Fluid
**pH**			
*on admission*	*7.56*	*8.5*	*5.16*
*1 month later*	*7.36*	*5.5*	*5.97*
**K^+ ^(mmol/l)**			
*on admission*	*2.6*	*113*	*18*
*1 month later*	*4.1*	*59*	*12*
**Na^+ ^(mmol/l)**			
*on admission*	*133*	*50*	*59*
*1 month later*	*140*	*25*	*53*
**Cl **^-^**(mmol/l)**			
*on admission*	*65*	*6*	*87*
*1 month later*	*109*	*85*	*46*

Upon admission the patient was rehydrated with physiologic saline solution at a rate of 200 ml/hour via a peripheral catheter. To each litre of infusion 20 mval potassium chloride were added. Fluid losses (stomal fluid and diuresis) averaged ~6.5 litres per day, oral fluid intake during the first days was in the range of 4-5 litres. In order to suppress gastric proton and chloride secretion the patient was treated with high dose proton pump inhibition (pantoprazole 8 mg/hour). Stool analysis did not reveal bacterial overgrowth.

**Figure 1:**
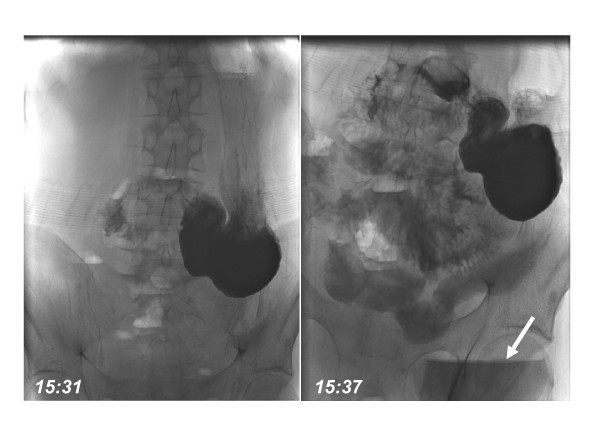
**Gastrografin enema**. Transit time from stomach to stoma (arrow) was 6 min.

An oral enema with gastrografin supported the tentative diagnosis of short bowel syndrome. The intestinal transit time from stomach to stoma was 6 minutes with no evidence for a gastrointestinal fistula (figure 1). Computertomography confirmed the diagnosis of short bowel syndrome. Due to excessive stomal fluid losses port implantation and nocturnal parenteral nutrition were initiated (1200 kcal).

Nasolabial dermatitis upon presentation was diagnosed as acrodermatitis enteropathica due to zinc deficiency (zinc level 23 μg/dl, normal range: 72-115, figure 2). Accordingly, the patient also received micronutrients and vitamins. Notably, vitamin B12 levels were normal at 803 pg/ml (normal range: 200-1100) indicating an intact terminal ileum.

**Figure 2:**
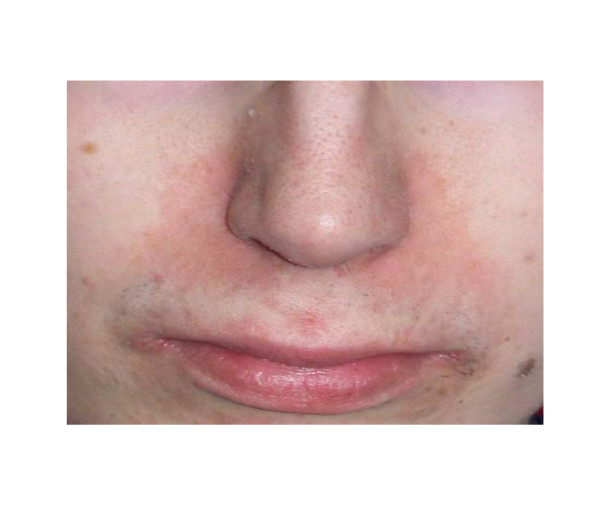
**Acrodermatitis enteropathica due to zinc deficiency**.

**Figure 3:**
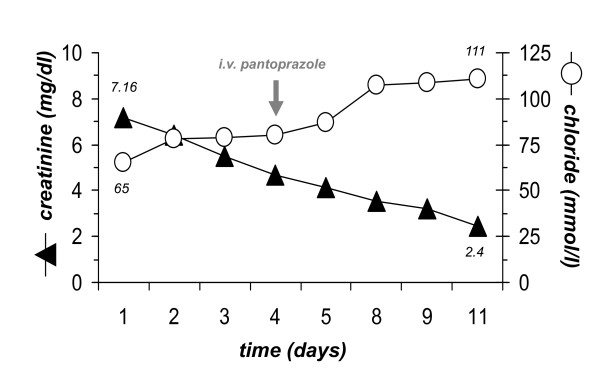
**Course of creatinine and chloride during volume replacement**.

Upon fluid replacement creatinine rapidly declined to 2.4 mg/dl (eGFR 32 ml/min) and thereby into the previously known range (figure 3). Initially, serum chloride levels slowly increased, administration of i.v. pantozole led to a rapid increase into the high normal range (figure 3). A follow-up arterial blood gas one week after admission showed marked improvement of acid-base homeostasis (pH 7.46, pO_2 _130 mmHg, pCO2 39.4 mmHg, bicarbonate 28 mmol/l, sodium 142 mmol/l, potassium 4.7 mmol/l, chloride 109 mmol/l, anion gap 5.0).

The patient was discharged in good condition after 11 days and advised to present at an intestine transplantation centre for evaluation of future combined intestinal and renal transplantation. At a follow-up visit 1 month later acid base and electrolyte imbalances had markedly improved (table 1), i.e. fecal chloride excretion was much lower at 46 mmol/l. Stomal fluid losses remained high at ~4000 ml/day.

## Conclusions

Severe metabolic alkalosis is a rare finding in patients with Crohn's disease. The most common acid-base disorder in these patients is metabolic acidosis due to D-Lactate uptake or gastrointestinal bicarbonate loss. In our patient metabolic alkalosis was triggered by gastrointestinal losses of volume and chloride, resulting in renal bicarbonate retention. Secondary hyperaldosteronism caused by volume depletion certainly contributed to metabolic alkalosis. However, gastrointestinal chloride loss (87 mmol/l) was particularly prominent. The acidic stool, the short intestinal transit time and the substantial improvement of chloride loss in response to proton pump inhibition all point to gastral HCl secretion. Intestinal inflammation may also lead to chloride secretion directly from the inflamed intestinal mucosa. Thus, inflammation reduces the intestinal expression of SLC26A3 [5] a chloride transporter that is mutated in patients with congenital chloridorrhea [6]. Acquired chloridorrhea has been described in patients with intestinal inflammation due to transplant rejection [7] and may be related to downregulation of SLC26A3 [5].

In our patient short bowel syndrome was associated with profound metabolic perturbations as evidenced by severe alterations in acid base homeostasis. Cornerstones in the treatment and management of severe metabolic alkalosis are adequate volume repletion as well as administration of potassium chloride since volume contraction, hypokalemia and hypochloremia are the key factors that maintain the condition. Volume contraction causes a rise in plasma bicarbonate concentration because there is contraction of the extracellular volume around a relatively constant quantity of extracellular bicarbonate [8]. Hypokalemia stimulates intracellular shift of hydrogen, in the kidney hypokalemia stimulates the luminally expressed V-type H^+^-ATPase of type A intercalated cells in the connecting tubules [9]. This results in proton secretion as well as bicarbonate reabsorption by basolateral chloride/bicarbonate exchangers [9]. Hypochloremia reduces chloride reabsoprtion via the SLC26A4 (pendrin) encoded Cl^-^/HCO3^- ^exchanger that is positioned on the apical membrane of type B intercalated cells [10]. This exchanger is tightly regulated and governs chloride and bicarbonate flux through the distal tubule. Consequently, the patient received large amounts of i.v. infusions together with potassium chloride.

Other therapeutic options in the management of metabolic alkalosis include blockade of bicarbonate reabsorption using carbonic anhydrase inhibitors. However, drugs such as acetazolamide promote potassium excretion by impeding hydrogen-linked sodium reabsorption [11]. This may aggravate hypokalemia and was therefore not considered in the present case. Antagonism of hyperaldosteronism using spironolactone may also be useful in treating metabolic alkalosis. Aldosterone stimulates H^+^-ATPase dependent bicarbonate reabsorption in all collecting duct segments of type A intercalated cells in the connecting tubules [12]. However, due to the patient's underlying chronic kidney disease and acute kidney injury treatment with spironolactone was not an option.

In cases of gastric hypersecretion H2-blockers as well as proton pump inhibitors or octreotide have been used to reduce stomal secretory losses which are in part triggered by lack of counteracting intestinal hormones such as gastric inhibitory polypeptide (GIP) or vasoactive intestinal polypeptide (VIP) [13]. Indeed, administration of pantozole hastened correction of hypochloremia in our patient even though stomal fluid losses remained substantial.

In severe cases of metabolic alkalosis the use of lysine or arginine hydrochloride may be considered. Considering the costs and side effects (hyperkalemia) these agents are currently not advocated [14]. Finally, hydrochloric acid has been used in humans to treat metabolic alkalosis, however such strategy requires utmost patient care, placement of a central venous catheter and close monitoring of arterial blood gases [15].

Other than that, patients are advised to small hourly oral feedings with food items high in complex carbohydrates [16]. In patients undergoing parenteral nutrition the initial formulation should guarantee adequate volume, electrolyte and micronutrient replacement. In recent studies, a combination of glutamine and growth hormone has been shown to hasten the adaptation process and enhance mucosal growth [17]. Furthermore, glucagon-like peptide 2 (GLP-2) has been implicated to improve nutrient absorption [18].

In case intestinal adaptation fails to develop, long-term parenteral nutrition is mandatory. Complications of parenteral nutrition include catheter infections or occlusions, venous thrombosis and progressive cholestatic liver disease that often requires combined intestinal and liver transplantation at later stages. Intestinal transplantation is reserved for patients who are either not candidates or have developed complications from long-term parenteral nutrition and in whom adequate adaptation does not occur.

In our case stomal fluid losses led to recurrent acute kidney injury necessitating implantation of a port catheter for parenteral nutrition. Stomal fluid losses of ~5000 ml per day were substantial in the light of normal stool volume which is in the range of 150 ml/day [19]. Under such conditions fecal electrolyte excretion is negligible (Na^+ ^20-30 mmol/l, K^+ ^55-75 mmol/l, Cl^- ^15-25 mmol/l) [19]. Due to the daily stool volume fecal chloride loss in our patient was markedly elevated and led to severe hypochloremia. Unfortunately, the arterial blood gas analyzer was unable to measure urinary or fecal bicarbonate concentrations. However, alkaluria (pH 8.5) suggests renal bicarbonate excretion as a compensatory mechanism in response to metabolic alkalosis. The latter was perpetuated by several mechanisms including volume contraction, hypokalemia, hypochloremia, secondary hyperaldosteronism and most importantly persisting acid losses via the stoma. In summary, our case highlights the complex nature of acid-base disturbances in patients with the short bowel syndrome.

## Consent

Written informed consent was obtained from the patient for publication of this case.

## Competing interests

The authors declare that they have no competing interests.

## Authors' contributions

JJ, SS, MO, KUE and KFH were the treating physicians of the patient reported. KA performed the evaluation of the renal biopsy. AK and AS performed the radiographic studies. The manuscript was prepared by JJ, all authors participated in the discussion of the manuscript and approved the final version.

## Pre-publication history

The pre-publication history for this paper can be accessed here:

http://www.biomedcentral.com/1471-2369/11/6/prepub
